# Changes in Noninvasive Liver Fibrosis Indices and Spleen Size During Chemotherapy: Potential Markers for Oxaliplatin-Induced Sinusoidal Obstruction Syndrome

**DOI:** 10.1097/MD.0000000000002454

**Published:** 2016-01-15

**Authors:** Sehhoon Park, Hwi Young Kim, Haeryoung Kim, Jin Hyun Park, Jung Ho Kim, Ki Hwan Kim, Won Kim, In Sil Choi, Yong Jin Jung, Jin-Soo Kim

**Affiliations:** From the Department of Internal Medicine (SP), Seoul National University Hospital, Seoul; Department of Internal Medicine (HYK, JHP, KHK, WK, ISC, YJJ, J-SK), Seoul National University Boramae Medical Center, Seoul; Department of Pathology (HK), Seoul National University Bundang Hospital, Bundang-gu, Seongnam; and Department of Pathology (JHK), Seoul National University Boramae Medical Center, Seoul, Korea.

## Abstract

Oxaliplatin-based regimens are standard treatments for the patients with colorectal cancer (CRC) and advanced gastric cancer (AGC). However, owing to hepatic sinusoidal obstruction syndrome (SOS), the use of oxaliplatin sometimes results in splenomegaly. The aim of the present study was to evaluate the correlation between chemotherapy-associated changes of noninvasive liver fibrosis indices and volumetric changes of the spleen.

From February 2004 to April 2014, patients with CRC or AGC receiving oxaliplatin-based chemotherapy were studied. The possibility of SOS development was evaluated before and after the oxaliplatin exposure with splenic volume index (SVI). Four different noninvasive liver fibrosis indices were used for risk analysis, namely age-platelet index (API), AST-to-platelet ratio index (APRI), platelet-to-spleen ratio (PSR), and fibrosis-4 score (FIB-4).

A total of 275 patients were eligible for evaluation: 200 patients had CRC and 75 patients had AGC. Using the cutoff of SVI increase ≥ 0.3, 113 patients (41.1%) were positive for splenomegaly. The changes of indices significantly correlated with SVI increase. Adjusted odds ratios for those indices were as follows: API = 1.16 (95% confidential interval [CI], 1.01–1.32; *P* = .03); APRI = 2.45 (95% CI, 1.30–4.63; *P* = .01); PSR = 0.69 (95% CI, 0.59–0.80; *P* < .01); and FIB-4 = 1.37 (95% CI, 1.16–1.63; *P* < .01). Optimal cutoff values with statistical significance were calculated and suggested.

The changes of noninvasive liver fibrosis indices showed a good correlation with the increase in the spleen volume during oxaliplatin-based chemotherapy. Validation of these indices for monitoring of oxaliplatin-induced hepatic SOS is warranted.

## Abstract 2

Supplemental Digital Content is available in the text

## INTRODUCTION

Oxaliplatin-based chemotherapy has been used for colorectal cancer (CRC) and advanced gastric cancer (AGC) patients in both adjuvant and palliative settings. The use of oxaliplatin has been increasing because of the high incidence rate of colorectal (71.3 per 100,000) and gastric (68.1 per 100,000) cancers in Korea.^1^ Examples of common oxaliplatin-based chemotherapy regimens for these cancers include FOLFOX (oxaliplatin, fluorouracil, and leucovorin), and XELOX (oxaliplatin and capecitabine).^2–6^ Both regimens are also applicable in the neoadjuvant setting for the patients with CRC with liver metastasis (CRCLM).^7,8^

However, various forms of chemotherapy-induced liver toxicity have been reported in the patients with or without underlying liver disease. Among those toxicities, sinusoidal obstruction syndrome (SOS) was first reported in 34 patients out of 43 patients (78%) had undergone hepatectomy for CRCLM after receiving oxaliplatin-containing chemotherapy.^9^ Owing to deposition of collagen and disruption of sinusoidal epithelium, liver specimens showed sinusoidal dilatation, hemorrhage, and veno-occlusion. Patients sometimes develop splenomegaly or other portal hypertension-related complications, which are similar to clinical features in the patients with liver cirrhosis.^10^ Currently, more and more patients are treated with oxaliplatin-based regimen in adjuvant setting of CRC and AGC. However, despite the anticancer benefit of oxaliplatin-based chemotherapy, SOS could be irreversible.^11,12^ Hence, early detection and possible prevention of SOS are crucial for these patients.

Although liver biopsy remains the gold standard for the diagnosis of SOS, the use of liver biopsy in practice has been limited because of its cost, potentially serious complications in rare cases, and concerns on sampling error, especially for asymptomatic patients.^13^ Owing to the lack of relevant research, little is currently known about the noninvasive prediction of oxaliplatin-induced SOS.^14,15^ Increase in the spleen size was reported to directly correlate with cumulative dosage of oxaliplatin.^16^ In a previous study, the spleen size increased in 86% of patients treated with FOLFOX regimen, with median increase in the spleen size amounting to 22%.^17^ Moreover, increased splenic volume (SV) was identified as a positive predictor of SOS.^16,18^ Recently, the role of noninvasive liver fibrosis indices^19^ has been actively investigated in the patients with various chronic liver diseases.^20–24^ These indices comprise various combinations of simple blood tests such as aspartate aminotransferase (AST), alanine aminotransferase (ALT), and platelet count (PLT).^25,26^ However, relevant studies on the role of noninvasive liver fibrosis indices in the early diagnosis of oxaliplatin-induced SOS are still scarce.

The aim of the present study was to elucidate the role of noninvasive liver fibrosis indices for the identification of presumed hepatic SOS by using volumetric changes of the spleen during oxaliplatin-based chemotherapy for the patients with CRC or AGC.

## PATIENTS AND METHODS

### Exploratory Dataset

Using our previous data with 89 CRCLM patients who received hepatic resection after chemotherapy as an exploratory dataset, we have performed a pilot study to evaluate the changes of noninvasive fibrosis indices and the spleen size based on oxaliplatin exposure during chemotherapy. Among these 89 patients, 23 patients were eligible for statistical analysis. The changes of the spleen size, age-platelet index (API), AST-to-platelet ratio index (APRI), fibrosis-4 score (FIB-4), platelet-to-spleen ratio (PSR), and histopathology were compared between the subjects with and without oxaliplatin exposure.^27^

### Study Population

The study patients’ baseline characteristics are shown in Table 1. From February 2004 to April 2014, a total of 412 patients met the following criteria in Seoul National University Boramae Medical Center: pathologically confirmed CRC and AGC, oxaliplatin-based chemotherapies (FOLFOX or XELOX) given as palliative or adjuvant chemotherapies. Because bevacizumab is known to be associated with a potential protective effect against hepatic SOS,^28,29^ the patients who received combination treatment with bevacizumab were excluded from the analysis (N = 8). The patients with positive serologic test for hepatitis B (N = 5) or C (N = 3) were excluded. None of the study subjects had previous history of alcoholic liver disease. In addition, the patients with insufficient laboratory values and imaging test were also excluded (N = 121). The final pool included 275 patients that were evaluated for the present study. This study was approved by the institutional ethics committee, and the requirements for informed consent from the patients were waived.

**TABLE 1:**
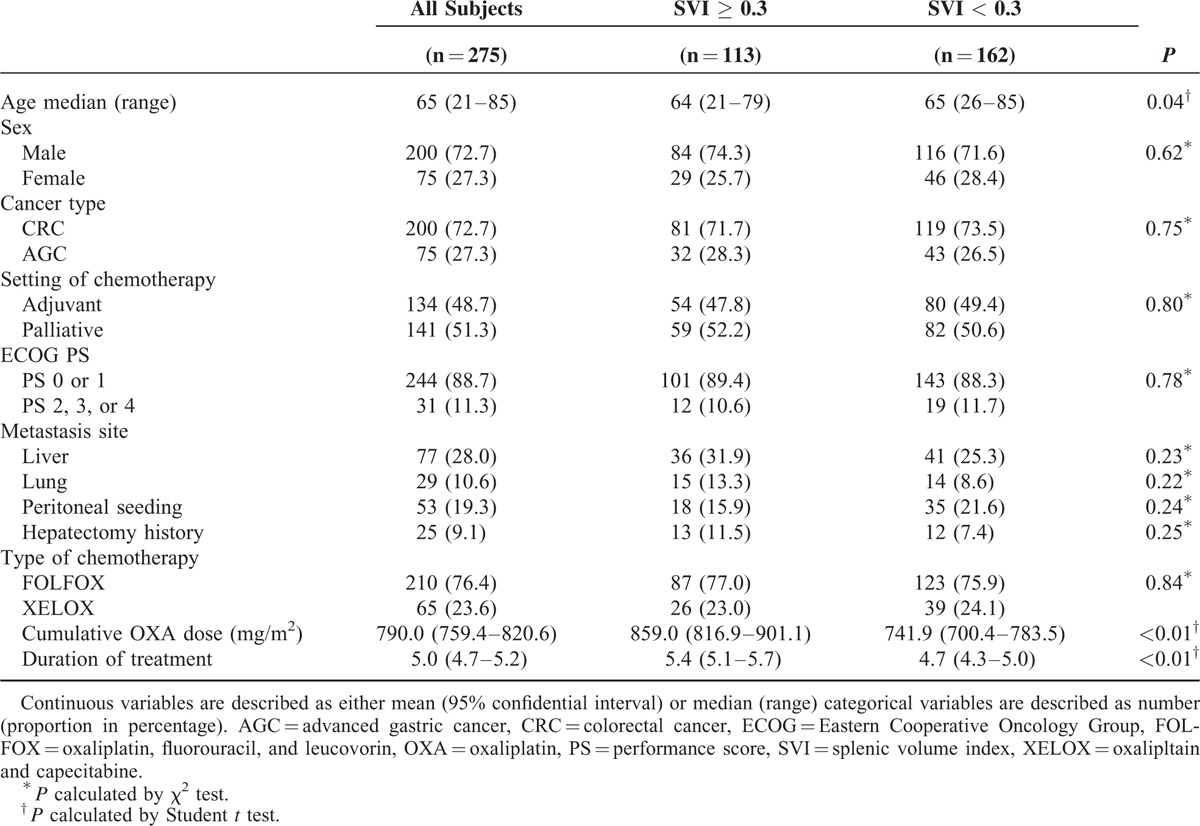
Comparison of Baseline Characteristic of Patients Treated With Oxaliplatin-Based Chemotherapy: All Patients and Patients Groups of High Versus Low Splenic Volume Index

### Data Collection

Routine laboratory tests, including blood cell counts and chemistries, were obtained before each cycle of chemotherapy. Computed tomography (CT) scans were performed every 2 to 3 cycles of chemotherapy. Baseline SVs were measured from the CT scans, and noninvasive liver fibrosis indices were calculated using blood test results before oxaliplatin-based chemotherapy. Cumulative oxaliplatin dosage was calculated. Based on hematologic or nonhematologic toxicities, dose reduction of oxaliplatin was allowed per physicians’ discretion. Surgical pathology specimens were reviewed from 4 patients who underwent tumorectomy of the liver after oxaliplatin-based chemotherapy.

### Calculation of Noninvasive Liver Fibrosis Indices and SV

Four noninvasive liver fibrosis indices were calculated as follows: API is the sum of age (<30 = 0; 30–39 = 1; 40–49 = 2; 50–59 = 3; 60–69 = 4; >70 = 5) and platelet count (PLT × 10^9^/L: ≥225 = 0; 200–224 = 1; 175–199 = 2; 150–174 = 3; 125–149 = 4; <125 = 5); APRI = [AST (× upper normal limit)/PLT (× 10^9^/L)] × 100; FIB-4 = [age (years) × AST (IU/L)]/[PLT (10^9^/L)] × [ALT (IU/L)^½^]; and PSR = PLT (× 10^9^/L)/spleen diameter (mm). Differences between the intervals are expressed as plus or minus values.^30–33^

SV was calculated by multiplying the width (mm), diameter (mm), and height of the spleen (mm) from the CT scan as described elsewhere.^6,16^ SV index (SVI) was determined by the following formula (1) and described in plus or minus values. The patients with SVI ≥ 0.3 were designated as “the high SVI” group, whereas those with SVI < 0.3 were “the low SVI” group.

 
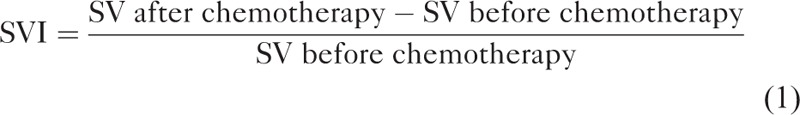


### Optimal Cutoff Value of Noninvasive Liver Indices

**FIGURE 2:**
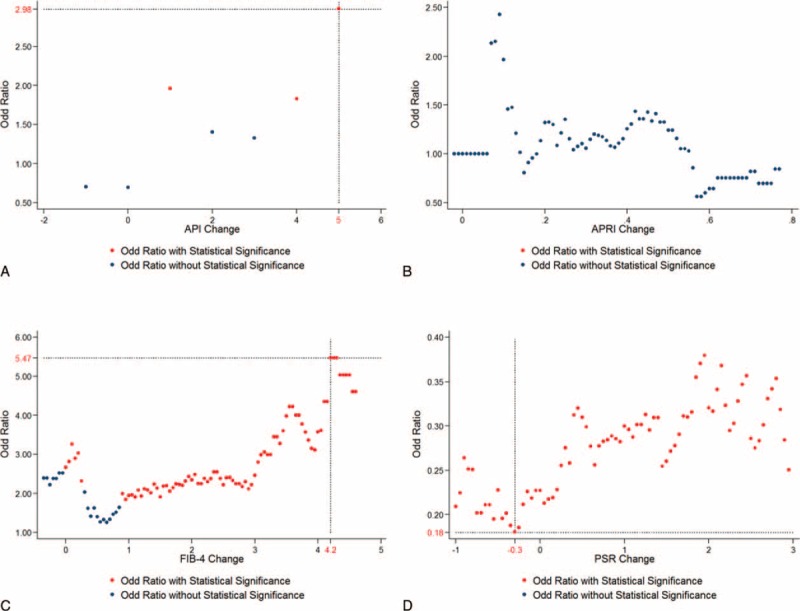
Odd ratio calculated by binary distribution using the difference of pretreatment and posttreatment values of (A) API, (B) APRI, (C) PSR, and (D) FIB-4. The patients were divided into 2 groups using the difference of pretreatment and posttreatment values. Each value from lower 10% to upper 10% was cut and odd ratios were calculated. All odd ratios were plotted in the figures and their statistical significance was marked (*P* < 0.05). API = age-platelet index, APRI = AST-to-platelet ratio index, FIB-4 = fibrosis-4 score, PSR = platelet-to-spleen ratio.

To calculate the optimal cutoff value, the recursive partitioning method was applied and odds ratio (OR) for the high SVI group was calculated and plotted.^34^ The recursive partitioning method was performed by calculating statistical significance with a potential cutoff value extracted by partitioning the changes of 4 noninvasive liver indices from lower 10% to upper 10%. The cutoff value with the highest OR which satisfied *P* value <0.05 is marked in Figure 2A–D.

### Statistical Analysis

Baseline characteristics of the study population were analyzed with descriptive statistics. The differences between the 2 groups were compared using Student *t* test. Categorical variables were evaluated with χ^2^ or Fisher exact tests. OR and adjusted odds ratio (aOR) for each variable were calculated with a multivariate logistic regression analysis. The variables with *P* ≤ 0.05 in univariate analyses and the variables with clinical relevance were included in the multivariate analysis. All statistical tests were 2-sided, *P* values <0.05 were considered as statistically significant. Statistical analyses were performed with IBM SPSS version 20.0 (SPSS Inc, Chicago, IL).

## RESULTS

### Characteristics of Exploratory Dataset

Among 23 patients with CRCLM, the patients treated with oxaliplatin-based chemotherapy (N = 11) were compared with the patients treated with non–oxaliplatin-based chemotherapy (N = 12). Changes of noninvasive liver fibrosis indices were more prominent in the former than in the latter group before hepatectomy. Focal hepatocyte injury (*P* = 0.04), parenchymal extrinsic lesion (*P* = 0.02), and ductal reaction (*P* = 0.04) were more frequently observed in the patients with oxaliplatin exposure (see Supplementary Table 1).

### Characteristics of Study Population

A total of 275 patients with oxaliplatin-treated CRC (N = 200, 72.7%) and AGC (N = 75, 27.3%) patients were evaluated. The patients were divided into 2 groups according to their SVI values (cutoff = 0.3): 113 patients (41.1%) in the high SVI group and 162 patients (58.9%) in the low SVI group. No statistical difference was observed between these 2 groups in terms of sex, type of cancer, Eastern Cooperative Oncology Group (ECOG) performance status (PS), site of metastasis, and other medical histories (see Table 1). However, the patients in the high SVI group were relatively younger than those in the low SVI group (*P* = 0.04). Most of the patients (N = 244, 88.7%) were in ECOG PS 0 and 1. FOLFOX was the first chemotherapy in 210 patients (76.4%). The high SVI group showed a significantly higher cumulative oxaliplatin dosage (*P* < 0.01) and a longer duration of treatment (*P* < 0.01) than the low SVI group.

### Changes of Noninvasive Liver Fibrosis Indices and SV

Based on the SVI with the cutoff value of 0.3, 4 noninvasive liver fibrosis indices (API, APRI, PSR, and FIB-4) were calculated at baseline and at each time point of response evaluation during the chemotherapy. All noninvasive liver fibrosis indices were significantly different compared with the baseline values in both groups after oxaliplatin exposure. However, when the absolute differences of these indices were compared, the changes were more prominent in the high SVI group than in the low SVI group (see Table 2). The distribution of changes (calculated by subtracting noninvasive index before the chemotherapy from index after it) for each noninvasive index is plotted in Supplementary Figure 1.

**TABLE 2:**
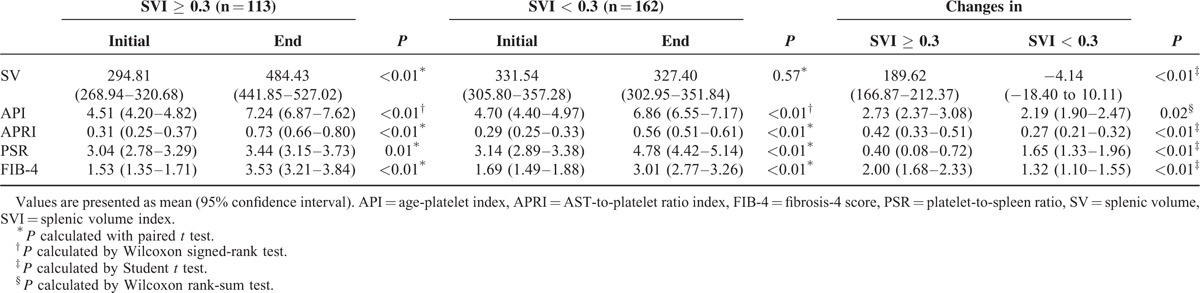
Distribution of Noninvasive Liver Fibrosis Indices for Patients With Splenic Volume Index ≥ 0.3 and Patients With Splenic Volume Index <0.3 After Chemotherapy

**FIGURE 1:**
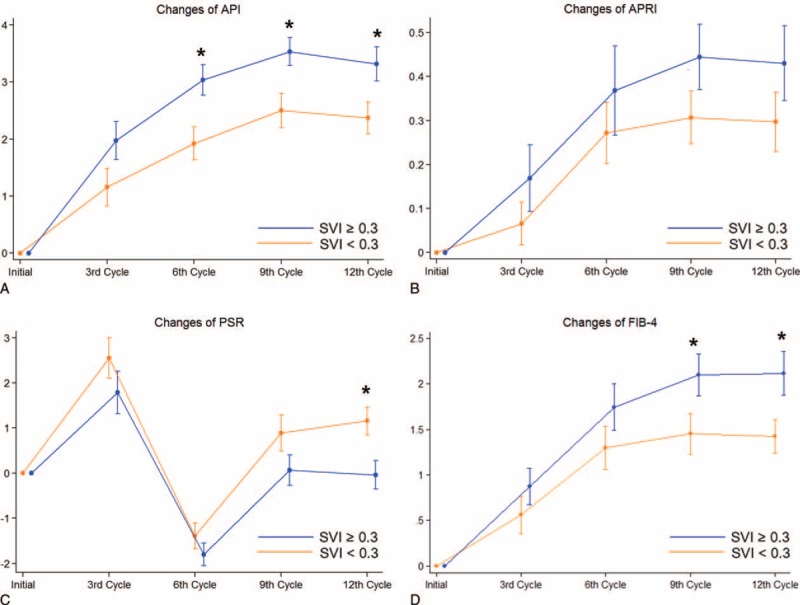
Sequential changes and standard error of the mean of noninvasive liver indices during 12 cycles of FOLFOX chemotherapy. Changes of 4 noninvasive liver indices: (A) API, (B) APRI, (C) PSR, and (D) FIB-4 were calculated at the initial 3rd, 6th, 9th, and 12th cycles. All patients were treated with 12 cycles of FOLFOX chemotherapy. ^∗^*P* < 0.05 calculated by the difference between the changes of indices as compared with baseline by the SVI group at each point. API = age-platelet index, APRI = AST-to-platelet ratio index, FIB-4 = gibrosis-4 score, PSR = platelet-to-spleen ratio.

Evolution of noninvasive liver fibrosis indices during chemotherapy was analyzed in 70 patients treated with 12 cycles of FOLFOX. Changes of noninvasive liver fibrosis indices and SVI were calculated at the point of the 3rd, 6th, 9th, and 12th cycles of chemotherapy. Changes of these indices between the high and low SVI groups were insignificant at the point of the 3rd cycle. However, following additional cycles of oxaliplatin-based chemotherapy, changes of API, PSR, and FIB-4 were statistically significant (see Fig. 1A, C, and D), except APRI (see Fig. 1B, *P* = 0.22). Unlike other noninvasive liver fibrosis indices, PSR increased at the early course of chemotherapy and decreased similar to the initial value with additional cycles (see Supplementary Table 2 and Figure 1C).

### Risk Factor Evaluation

Using the cutoff of SVI ≥ 0.3, all of the 4 noninvasive liver fibrosis indices significantly correlated with SVI. ORs for changes in those indices were as follows: 1.17 for API (95% CI, 1.02–1.33, *P* = 0.02), 2.48 for APRI (95% CI, 1.30–4.63, *P* < 0.01), 0.68 for PSR (95% CI, 0.58–0.80, *P* < 0.01), and 1.33 for FIB-4 (95% CI, 1.13–1.56, *P* < 0.01). Age was also significantly associated with SVI (OR 0.98, 95% CI, 0.96–1.00, *P* = 0.05). After adjustment for age, type of cancer, and chemotherapy setting, the adjusted ORs for each liver fibrosis indices significantly associated with SVI increase (see Table 3).

**TABLE 3:**
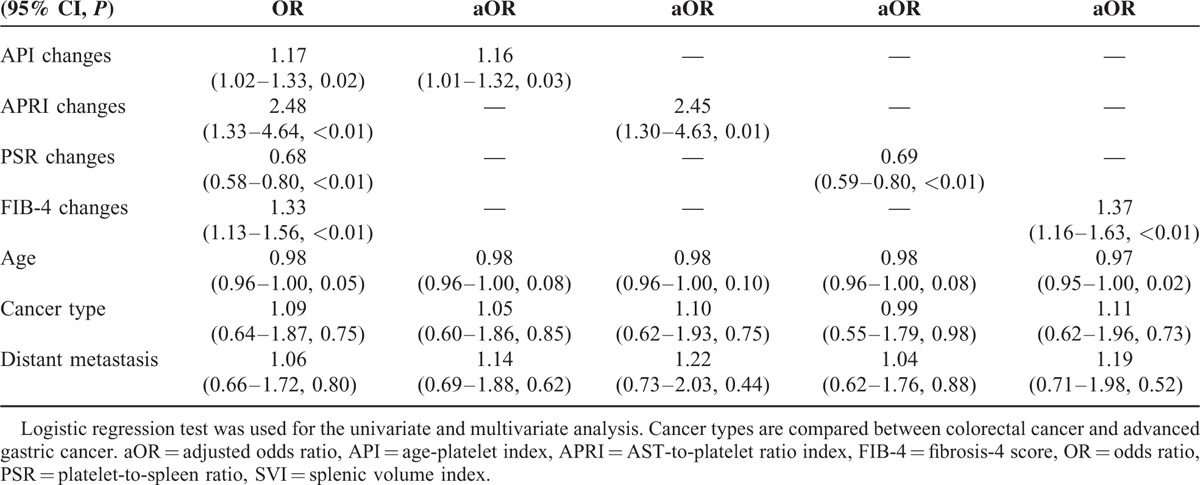
Univariate and Multivariate Analysis of Noninvasive Liver Fibrosis Indices and Factors Contributing SVI ≥ 0.3 after OXA-Based Chemotherapy

### Optimal Cutoff Value of Noninvasive Liver Indices

API showed the highest OR of 1.98 (95% CI, 1.05–2.69, *P* = 0.03). Similarly, PSR change of −0.3 was the optimal cutoff value with the highest risk reduction (OR 0.21, 95% CI, 0.11–0.39, *P* < 0.01) and FIB-4 change of 3.55 satisfied statistical significance with the highest OR 4.39 (95% CI, 1.87–10.32, *P* < 0.01). However, APRI change did not reach statistical significance. The distribution of ORs and their statistical significance are shown in Figure 2A–D.

### Pathologic Evaluation of Surgical Specimens

Pathologic changes of the liver after oxaliplatin exposure were evaluable in 4 patients. The characteristics of these patients and noninvasive liver fibrosis indices are described in Supplementary Table 3. Two patients were in the high SVI group, and 1 of them showed grade 2 of sinusoidal dilatation. The other 2 patients in the low SVI group showed grade 1 hepatosinusoidal injury. Representative photomicrographs of patient 1 are shown in Supplementary Figure 2. Among the 4 patients, 1 patient (patient 4) experienced several symptoms related to portal hypertension after hepatic resection. The detailed clinical course of patient 4 is described in Supplementary Table 3 and serial CT scans (pretreatment, postchemotherapy, postliver resection); esophagogastroduodenoscopy pictures demonstrating esophageal varices are shown in Supplementary Figure 3.

## DISCUSSION

The aim of the present study was to evaluate potential usefulness of currently available noninvasive biomarkers of liver fibrosis for early detection of presumed hepatic SOS by volumetric changes of the spleen during oxaliplatin-based chemotherapy. Our results demonstrate that the evolution of noninvasive liver fibrosis indices during oxaliplatin-based chemotherapy is significantly associated with increase in SVI, which is known as one of the noninvasive markers for hepatic SOS. Changes of noninvasive liver fibrosis indices were more prominent in the high SVI group. Thus, significant increases of theses indices might represent an increased risk of the development of SOS.

Oxaliplatin-based chemotherapy has been widely used as a standard adjuvant chemotherapeutic regimen for CRC and AGC patients.^3,35^ The risk of hepatic complications is elevated after hepatectomy, if more cycles of preoperative chemotherapies are given.^8,36^ Although early detection of SOS may improve the quality of life, prediction of SOS using noninvasive liver fibrosis indices has not been extensively studied yet. Miura et al^19^ suggested that aspartate aminotransferase-to-platelet ratio (APR) and increases in the spleen size might be useful in predicting SOS in the patients with CRC treated with FOLFOX. However, the number of patients referred to the study was relatively small (N = 71), and changes during the chemotherapy were not evaluated.^19^ In the present study, changes of 4 noninvasive liver fibrosis indices during oxaliplatin-based chemotherapy were evaluated in a relatively larger patient population (N = 275). Cutoff values were determined with the manual approach of cutting the difference of each noninvasive liver index in a short interval. The risk of SOS was considered as high when API changes ≥5, or FIB-4 changes ≥4.2 during oxaliplatin-based chemotherapy. In the same manner, PSR change <−0.3 could be considered as high risk for SOS. These noninvasive indices can be easily accessible, and applicable markers for the patients with oxaliplatin exposure who show volumetric changes of the spleen. In particular, these cutoff values might be helpful in the decision regarding performing liver biopsy and operability in the patients who are candidate for liver resection with curative intent. However, further studies are needed to determine comparability of specific indices in their accuracies to rule in or out the presence of SOS.

Although liver biopsy is the standard method for the diagnosis of SOS, its limitations—such as the cost, invasiveness, and the possibility of sampling error and interobserver variability—are also well known.^37^ By contrast, noninvasive liver fibrosis indices have a strong advantage in terms of safety, cost, and patient compliance. Components of the noninvasive liver fibrosis indices are based on AST, ALT, PLT count, and diameter of the spleen, all of which are easily acquired from routine blood tests and imaging work-up during regular follow-up. Because, owing to its aforementioned limitations, liver biopsy cannot be routinely recommended for early detection of SOS in asymptomatic patients, the necessity of noninvasive method for early prediction was raised. In fact, we were able to identify only 89 patients whose liver specimen after chemotherapy was available from 2003 to 2009 in the previous report from Seoul National University Bundang Hospital; among them, 11 patients were exposed to oxaliplatin-based chemotherapy. Because there was a small number of patients with available liver biopsy after oxaliplatin exposure, we used the spleen size as a surrogate marker of SOS, as demonstrated in a previous study.^16^ Using volumetric changes of the spleen as a marker, additional hepatic pathologic changes were confirmed by a direct comparison between the groups with SVI ≥ 0.3 and SVI < 0.3. Grades 2 or 3 of sinusoidal injury were more common in the group with high SVI than in the group with low SVI (*P* = 0.02).^19^ Increase in the spleen size also significantly correlated with accumulated oxaliplatin dose in our study population (data not shown), and sinusoidal injury was shown in the patient in the high SVI group. Among 4 patients who underwent hepatic resection after oxaliplatin-based chemotherapy, surgical specimens of a patient in the high SVI group showed all the characteristics of SOS, including small vessel obliteration, centrilobular vein fibrosis, and moderate sinusoidal dilatation (Supplementary Table 3 and Figure 2). Based on these observations, the cutoff value of SVI ≥ 0.3 was used for the definition of hepatic SOS in the present study.

The present study has several limitations. First, acquisition of liver histopathologic data in a small portion of the patients prevented a direct extrapolation of the results to the prediction of SOS. Second, regarding limited statistical power from a single-cohort patient population, the optimal cutoff value with statistical significance was not achievable in 1 out of 4 noninvasive liver fibrosis indices. Third, although correlation between volumetric changes of the spleen and SOS is well established, several studies have shown a direct correlation between SOS and SVI, which was a surrogated marker of SOS in the present study.

In conclusion, changes of noninvasive liver fibrosis indices during oxaliplatin-based chemotherapy showed a good correlation between volumetric changes of the spleen size during chemotherapy. Thus, they might serve as good predictive markers for SOS, especially APRI. Given the noninvasive property and availability in daily practice, these markers might be used for SOS monitoring in the majority of cases during oxaliplatin-based chemotherapy after a proper validation in a large-scale prospective study with a histological comparison.

## Supplementary Material

Supplemental Digital Content
