# Supplementary material for: Changes in Noninvasive Liver Fibrosis Indices and Spleen Size During Chemotherapy: Potential Markers for Oxaliplatin-Induced Sinusoidal Obstruction Syndrome
Source: Medicine (Baltimore). 2016 Jan 15;95(2):e2454. doi: 10.1097/MD.0000000000002454 (PMC4718264; doi:10.1097/MD.0000000000002454)

Title: Changes in Non-invasive Liver Fibrosis Indices and Spleen Size During Chemotherapy: Potential Marker for Oxaliplatin-Induced Sinusoidal Obstruction Syndrome

Authors: Sehhoon Park, MD, Hwi Young Kim, MD, PhD, Haeryoung Kim, MD, PhD, Jin Hyun Park, MD, Jung Ho Kim, MD, PhD, Ki Hwan Kim, MD, PhD, Won Kim, MD, PhD, In Sil Choi, MD, PhD, Yong Jin Jung, MD, PhD, Jin-Soo Kim, MD, PhD

Supplementary Table 1. Correlation of non-invasive fibrosis indices and the characteristics of pathologic findings from the previous publication.

	Oxaliplatin-based chemotherapy (n=11, 47.8%)	No oxaliplatin-based chemotherapy (n=12, 52.2%)	*P*
SVI			
≥ 0.3	4 (36.4)	2 (16.7)	.37[a]
< 0.3	7 (63.6)	10 (83.3)	
API change	71.6 (-23.5-166.7)	23.6 (-26.8-73.9)	.32[b]
APRI change	0.45 (0.15-0.74)	0.23 (-0.5-0.51)	.25[b]
PSR change	1.44 (0.52-2.37)	2.83 (1.15-4.51)	.14[b]
FIB-4 change	1.43 (0.62-2.25)	0.78 (-0.23-1.79)	.28[b]
Small vessel obliterans	9 (81.8)	5 (41.7)	.09[a]
Focal hepatocyte injury	9 (81.8)	4 (33.3)	.04[a]
Parenchymal extrinsic lesion	6 (54.6)	1 (8.3)	.02[a]
NRH	1 (9.1)	0 (0.0)	.48[a]
Sinusoidal dilatation			
Grade 0	1 (9.1)	5 (41.7)	.07[a]
Grade 1	7 (63.6)	7 (58.3)	
Grade 2	3 (27.3)	0 (0.0)	
Centrilobular vein fibrosis	8 (72.7)	6 (50.0)	.25[a]
Macrovesicular steatosis			
Grade 0	5 (45.5)	6 (50.0)	1.00[a]
Grade 1	4 (36.4)	5 (41.7)	
Grade 2	2 (18.2)	1 (8.3)	
Microvesicular steatosis			
Grade 0	6 (54.5)	7 (58.3)	1.00[a]
Grade 1	4 (36.4)	5 (41.7)	
Grade 2	1 (9.1)	0 (0.0)	
Hepatocyte ballooning	3 (27.3)	3 (25.0)	1.00[a]
Fibrosis			
Periportal	1 (9.1)	4 (33.3)	.72[a]
Portal	4 (36.4)	3 (25.0)	
Bridging	5 (45.4)	4 (33.3)	
Cirrhosis	1 (9.1)	1 (8.3)	
Cholestasis	3 (27.3)	1 (8.3)	.32[a]
Portal lymphocyte infiltration			
Grade 0	4 (36.4)	5 (41.7)	1.00[a]

Grade 1	6 (54.6)	6 (50.0)	
Grade 2	1 (9.1)	0 (0.0)	
Grade 3	0 (0.0)	1 (8.3)	
Ductal reaction	7 (63.7)	2 (16.7)	.04[a]

Oxaliplatin-based regimen: FOLFOX, XELOX
No oxaliplatin-based regimen: Capecitabine single, 5-FU and leucovorin, FOLFIRI, UFTE, UFTE and leucovorin
[a] *P* calculated by Fisher's exact test
[b] *P* calculated by Student's t-test
Abbreviations: NRH = nodular regeneration hyperplasia, FOLFOX = oxaliplatin, fluorouracil and leucovorin, XELOX = capecitabine and oxaliplatin, FOFIRI = irinotecan, fluorouracil and leucovorin

Supplementary Table 2. Sequential changes of non-invasive liver fibrosis indices in the 70 patients treated with 12 cycles of FOLFOX

Mean (95% CI)	SVI ≥ 0.3 (n=32)	SVI <0.3 (n=38)	*P*	SVI ≥0.3 (n=32)	SVI <0.3 (n=38)	*P*
	3rd cycle of FOLFOX			6th cycle of FOLFOX		
SVI	0.17 (0.06-0.29)	-0.94 (-0.16 to -0.03)	<.01[a]	0.33 (0.21-0.44)	-0.03 (-0.12- 0.06)	<.01[a]
API changes	1.97 (1.29-2.64)	1.16 (0.48-1.84)	.09[a]	3.03 (2.49-3.58)	1.92 (1.33-2.51)	.01[a]
APRI changes	0.17 (0.01-0.32)	0.07 (-0.03 to 0.16)	.11[b]	0.37 (0.16-0.58)	0.27 (0.13-0.41)	.23[b]
PSR changes	1.49 (0.83-2.75)	2.55 (1.65-3.46)	.24[a]	-1.80 (-2.32 to -1.29)	-1.39 (-1.97 to -0.81)	.29[a]
FIB-4 changes	0.87 (0.46-1.29)	0.56 (0.14-0.98)	.29[a]	1.74 (1.22-2.26)	1.29 (0.81-1.78)	.21[a]
	9th cycle of FOLFOX			12th cycle of FOLFOX		
SVI	0.47 (0.36-0.58)	0.07 (-0.04 to 0.19)	<0.01[a]	0.73 (-0.62-0.83)	0.01 (-0.06 to 0.08)	<.01[a]
API changes	3.53 (3.03-4.03)	2.50 (1.89-3.11)	.01[a]	3.31 (2.70-3.92)	2.37 (1.80-2.93)	.02[a]
APRI changes	0.44 (0.29-0.60)	0.31 (0.19-0.43)	.12[b]	0.43 (0.26-0.60)	0.30 (0.16-0.43)	.05[b]
PSR changes	0.07 (-0.62 to 0.75)	0.89 (0.07-1.70)	.13[a]	-0.04 (-0.69 to 0.61)	1.16 (0.53-1.78)	.01[a]
FIB-4 changes	2.10 (1.63-2.56)	1.45 (1.00-1.90)	.05[a]	2.11 (1.63-2.60)	1.42 (1.04-1.80)	.03[a]

Patient was categorized into two groups, SVI≥ 0.3 and SVI<0.3 which were calculated at the end of chemotherapy. Changes were calculated by subtracting value at beginning of chemotherapy from value at each cycle. [a] *P* calculated by Student's t-test; [b] *P* calculated by Wilcoxon rank-sum test
Abbreviations: SVI = splenic volume index, SV = splenic volume, API = age-platelet index, APRI = AST to platelet ratio index, PSR = platelet to spleen ratio, FIB-4 = fibrosis-4 score, FOLFOX = oxaliplatin, fluorouracil and leucovorin

Supplementary Table 3. Pathologic characteristics of 4 patients who underwent hepatectomy after treated with oxaliplatin based chemotherapy in Boramae Medical Center

	Patient 1	Patient 2	Patient 3	Patient 4
SVI	0.62	0.22	0.02	1.25
Age	50	70	72	66
Sex	Male	Male	Male	Male
Type of cancer	Colon cancer	Colon cancer	Colon cancer	Gastric Cancer
Initial stage	4	4	4	4
Cumulative oxaliplatin dose (mg/m^2)	255	510	765	1040
API change	1	3	4	5
APRI change	0.378	0.277	0.847	0.523
PSR change	-0.570	2.220	-0.270	-0.892
FIB-4 change	1.469	0.724	2.325	3.798
Sinusoidal dilatation	Grade 2 (moderate)	Grade 1 (mild)	Grade 1 (mild)	Grade 1 (mild)
Centrilobular vein fibrosis	Present	Present	NA	Absent
Small vessel obliteration	Present	Absent	NA	Absent
Hepatocyte plate disruption	Present	Present	Present	Present
Parenchymal extinction lesion	Present	Present	NA	Present

Medical history of patient 4. A 66 year-old male patient presented with advanced gastric adenocarcinoma with a metastatic lesion in the left lobe of liver. He had no history of chronic liver disease and showed no sign of portal hypertension at initial evaluation. Pretreatment Child- Pugh Score was 5, and non-invasive liver fibrosis indices were as follows: API was 4; APRI was 0.124; PSR was 5.200; FIB-4 was 0.942. A total of 8 cycles of XELOX (oxaliplatin – 130mg/m2 at day 1 and capecitabine – 1000mg/m2 at day 1 to day 14) were given every 3 weeks. Best objective response was a partial response by RECIST 1.1. After the chemotherapy, Child-Pugh Score was 6, and non-invasive liver fibrosis indices were as follows: API was 9; APRI was 0.647; PSR was 4.308; FIB-4 was 4.740. Splenic volume index was 1.25. Subtotal gastrectomy with left lateral liver sectionectomy was performed at 5th week after the last dose of chemotherapy. At 2 months after the surgery, the patient complained of abdominal distension. CT scans and endoscopic examination revealed ascites and grade I esophageal varix with Child-Pugh Score of 8 (Child-Pugh class B)

Abbreviations: SVI = splenic volume index, SV = splenic volume, API = age-platelet index, APRI = AST to platelet ratio index, PSR = platelet to spleen ratio, FIB-4 = fibrosis-4 score

Supplementary Fig 1. Distribution of changes of (A) SVI, (B) API, (C) APRI, (D) PSR, and (E) FIB-4

Changes of each index were calculated by subtracting the index value after the chemotherapy from the index value before the chemotherapy. All actual values were plotted.

Abbreviations: SVI = splenic volume index, SV = splenic volume, API = age-platelet index, APRI = AST-to-platelet ratio index, PSR = platelet-to-spleen ratio, FIB-4 = Fibrosis-4 score

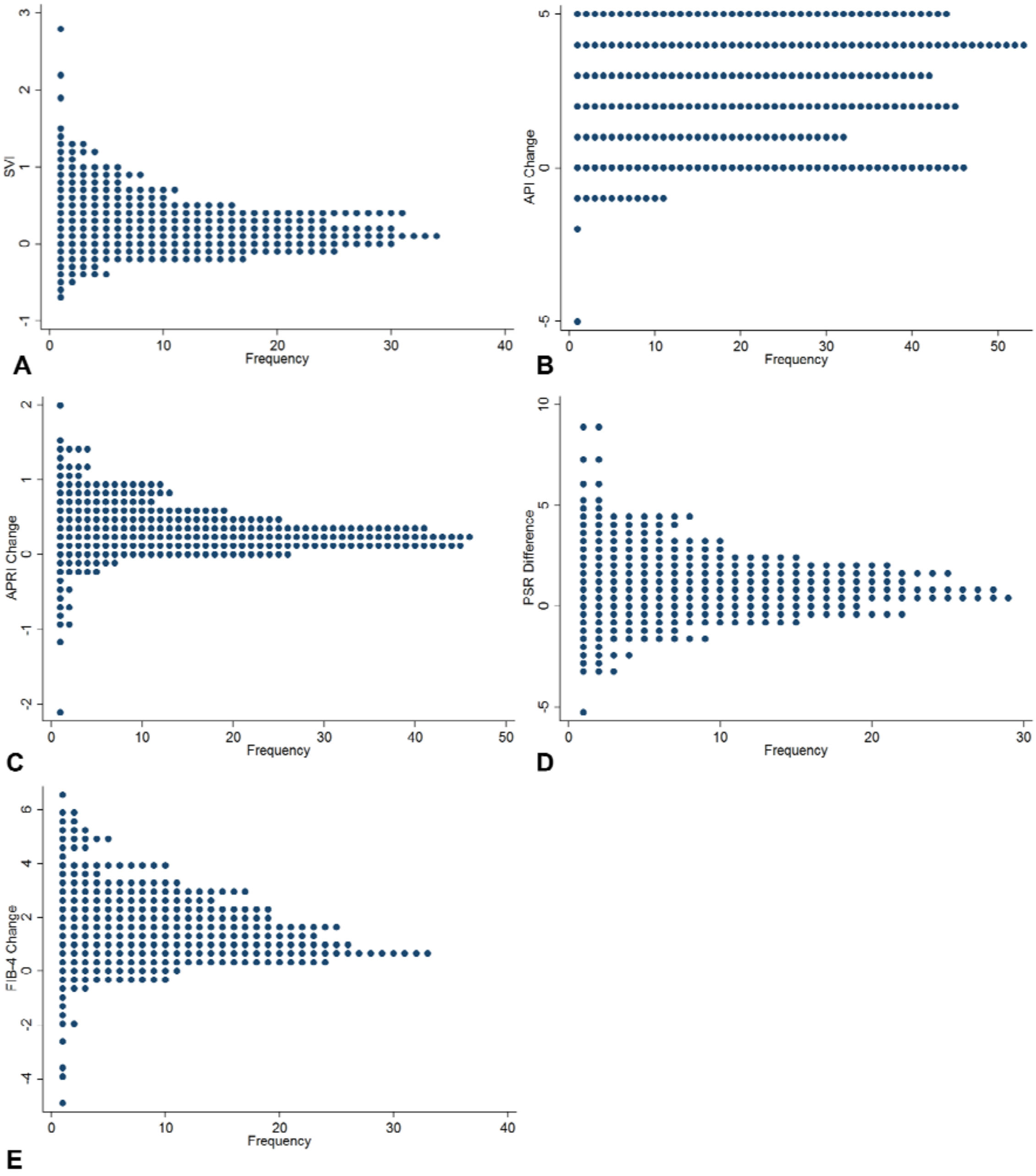

Supplementary Fig. 2. Photomicrographs of the histopathologic finding of patient 1 in Supplementary Table 3.

(A) Centrilobular fibrosis and small vessel obliterans (X200), (B) Sinusoidal dilatation and hepatocyte plate disruption (X100), (C, D) Sinusoidal dilatation and parenchymal extinction lesion (X50)

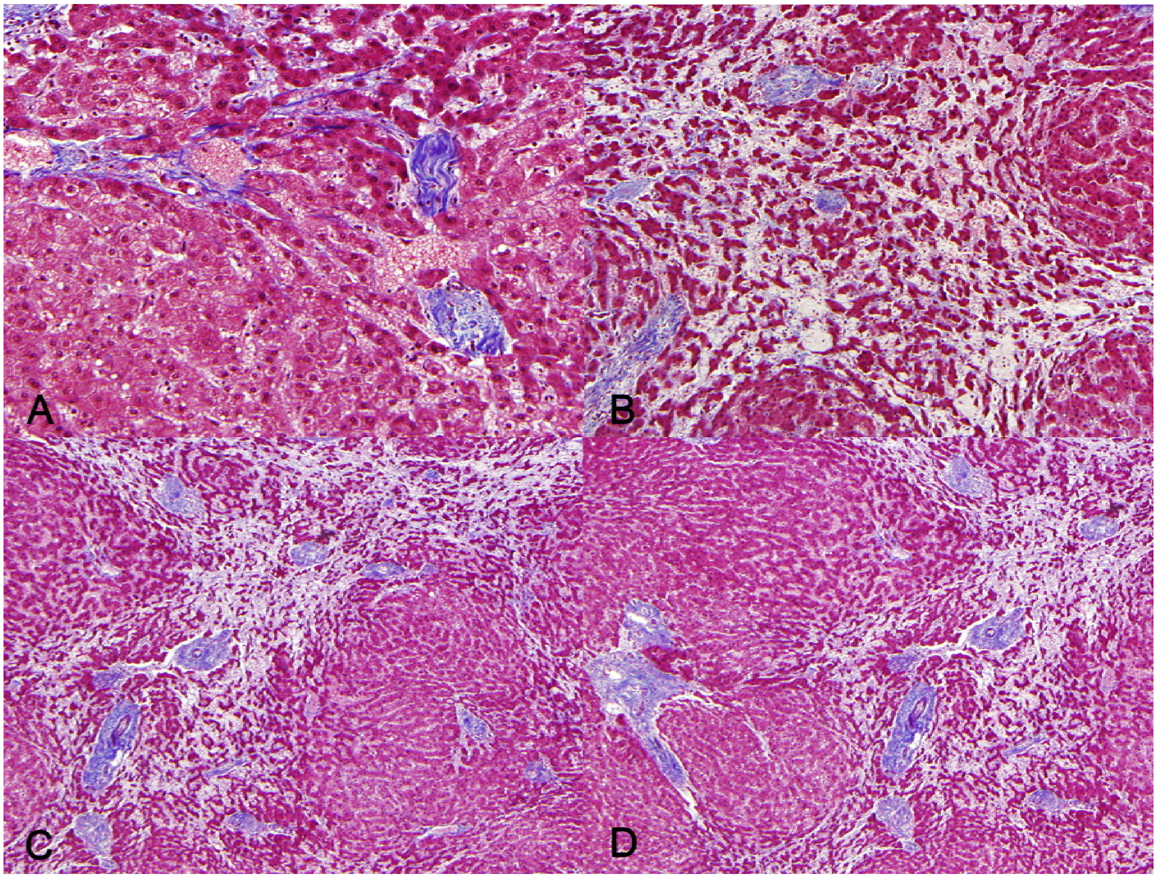

Supplementary Fig. 3. CT scans and endoscopic picture of esophageal varix of patient 4 in Supplementary Table 3.

(A) Pretreatment CT scans show advanced gastric cancer with a 1.2 cm-sized metastatic lesion in liver, (B) After 8 cycles of XELOX chemotherapy, CT images show a partial response with decreased liver metastasis and gastric lesion. Note that the spleen size increased compared to the one shown in Figure A. (C) 2 months after subtotal gastrectomy with the left lateral liver sectionectomy, the CT scan shows a moderate amount of ascites with a diffuse edematous wall thickening of the bowel walls. (D) Endoscopic picture at 2 months after surgery shows linear mild venous distensions with grade 1 esophageal varix in the distal esophagus.

Abbreviations: XELOX = oxaliplatin and capecitabine; CT = computed tomography

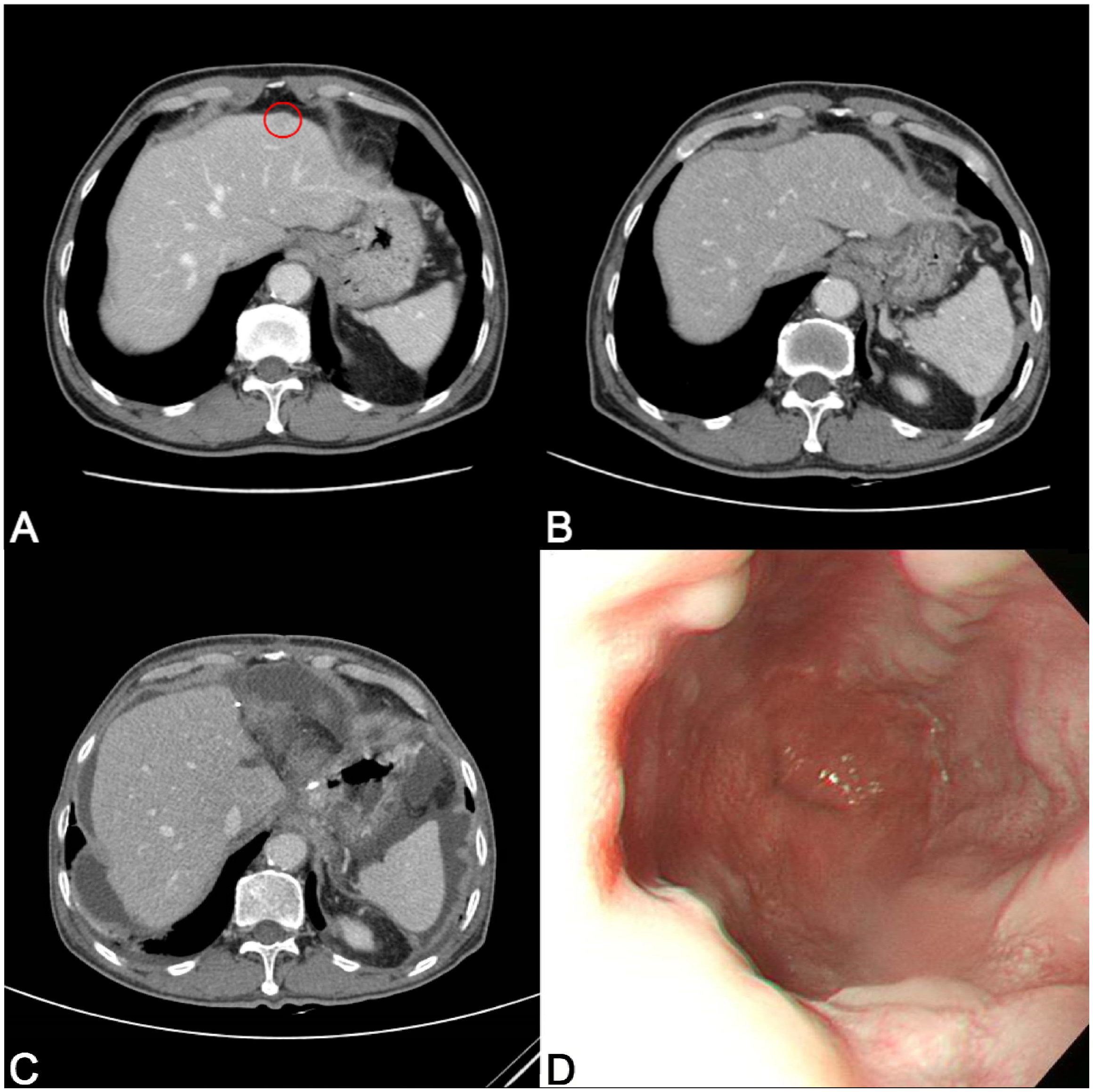

Supplement: Supplemental Digital Content [file medi-95-e2454-s001.pdf]
